# Editorial: Advances in clinical neuropsychology and interplay with mental health in several health conditions

**DOI:** 10.3389/fpsyt.2026.1839115

**Published:** 2026-04-10

**Authors:** Eduardo Fernández-Jiménez, Stephen L. Aita, Robert M. Roth

**Affiliations:** 1 Faculty of Law, Education and Humanities, Universidad Europea de Madrid, Madrid, Spain; 2 Department of Child and Adolescent Psychiatry, Clinical Psychology and Mental Health, La Paz University Hospital, Madrid, Spain; 3 Hospital La Paz Institute for Health Research (IdiPAZ), Madrid, Spain; 4 Department of Neurology, Whiddon College of Medicine, University of South Alabama, Mobile, AL, United States; 5 Dartmouth College Geisel School of Medicine, Hanover, NH, United States

**Keywords:** clinical neuropsychology, editorial, health conditions, interdisciplinarity, mental health

## Introduction

Clinical neuropsychology is an applied neuroscientific discipline that increasingly integrates complex neurobiological data with ecologically valid behavioral information in clinical practice. We are transitioning toward a more stratified and dimensional understanding of mental health conditions, based on continuous spectra, following the precision medicine framework (1). Moreover, digital assessment technologies (2) and artificial intelligence models (3–5) are revolutionizing the field. In this context, the present Research Topic published in Frontiers in Psychiatry provides diverse insights into neurodevelopmental, mental and neurocognitive disorders, along with innovative treatment approaches, bridging the gap between neuropsychology and clinical practice. This Editorial integrates 10 recent contributions, focused on the interplay between neurocognitive outcomes and various health conditions, encompassing reformulated theoretical frameworks, updated reviews, as well as original observational studies and randomized controlled trials.

An interesting paradigm shift is introduced through the framework of systemic neuropsychology. Guiral conceptualized psychological functions as “Topological Functional Systems” and proposed a theoretical and mathematical formalization concerning psychological activity, emphasizing its systemic and dynamic organization, as well as its adaptive function. This theoretical advancement has relevant clinical implications for neuropsychological assessment and rehabilitation, particularly for the management of complex disorders.

Within the spectrum of neurodevelopmental conditions, researchers are refining our understanding of Attention-Deficit/Hyperactivity Disorder (ADHD) by examining multidimensional factors associated with core symptoms. In this sense, the study by Fu et al. suggests that ADHD symptomatology follows differential developmental trajectories and is influenced by specific phenotypes. In particular, using the Swanson, Nolan, and Pelham Rating Scale-IV (SNAP-IV), they found that inattention remained stable across age and sex, and is more linked to learning problems, whereas hyperactivity-impulsivity is more related to younger age, boys, conduct problems, sleep-wake transition disturbances, and lower levels of anxiety. Furthermore, the work of Pan et al. on the validation of the SNAP-IV in amblyopic children highlights the relevance of population-specific assessment protocols to prevent diagnostic misattribution and ensure early intervention in visually impaired youth.

Notably, significant progress has been made in research on schizophrenia endophenotypes. These outcomes serve as intermediate and mediating variables between polygenic risk and clinical outcomes. In particular, a recent review by Garcia et al. highlighted that while oculomotor, event-related potentials, and cognitive deficits are consistently considered established endophenotypes in people living with schizophrenia, social cognition components, such as Theory of Mind and emotion processing, are emerging as promising candidate endophenotypes. These findings foster a shift toward “stratified psychiatry”, aiming to improve remission rates by matching patients to interventions based on specific endophenotype profiles. In the same direction, Øie et al. also emphasized the relevance of neuropsychological assessments and individualized rehabilitation in this population, highlighting the role of clinical neuropsychology in schizophrenia care regarding several areas.

From a long-term perspective, longitudinal studies on verbal memory in patients with major depressive disorder (MDD) offer a more optimistic view of cognitive trends than has been reported previously. In particular, Schmid et al. studied patients with a first depressive episode during five-year follow-ups and found that auditory attention was impaired during the acute phases, whereas verbal memory performance normalized to the level of healthy controls during remission. However, deficits in auditory attention may negatively affect daily memory due to its reliance on attentional processes. Indeed, attention deficits are often subjectively reported by patients experiencing a depressive episode.

Beyond primary mental health disorders, the intersection between physical health and psychological morbidity represents another significant area of research. İlhan et al. presented the clinical complexity of Hashimoto’s encephalopathy among Turkish female patients hospitalized due to mental health disorders, underscoring the diagnostic challenges when neuro-inflammatory disorders mimic primary mental health conditions. Accordingly, identifying “red-flag” signs, such as the delirium-catatonia syndrome, is essential for the timely initiation of corticosteroid therapy, which can yield rapid and substantial remission in cases otherwise refractory to standard psychopharmacological interventions.

Similarly, the close association between acute delirium and posttraumatic stress disorder (PTSD) has been examined through a meta-analysis conducted by Yang et al., showing that patients with delirium faced a significantly higher risk (adjusted odds ratio = 3.96) of developing subsequent PTSD. Therefore, this finding indicates that delirium is not merely a transient state but a potential precursor to long-term psychological impairment, likely mediated by the formation of delusional memories during acute brain dysfunction. Likewise, the relevance of identifying psychopathological signs is further supported by rare case reports, such as the one described by Alhaj et al. regarding intussusception caused by pica in a 13-year-old Middle Eastern girl. In this case, the persistent ingestion of foreign objects served as a maladaptive coping strategy for a severe comorbid MDD, ultimately requiring urgent laparotomy.

Moreover, advancements in neurostimulation provide hope for improving outcomes in both acute and chronic conditions. In particular, in a randomized controlled trial conducted by Xia et al., intermittent theta-burst stimulation (iTBS) of the left dorsolateral prefrontal cortex, combined with cognitive training, proved efficacious in enhancing global cognition, executive function, and working memory in patients with post-stroke cognitive impairment. Importantly, these cognitive gains correlated with significant reductions in serum lactate dehydrogenase levels, suggesting that iTBS may have potent anti-inflammatory or neuroprotective effects.

## Conclusion

This Research Topic addresses updated applications of clinical neuropsychology to several health conditions, including both medical illnesses and mental disorders. The 10 studies included in this Research Topic demonstrate that clinical neuropsychology is a neuroscientific discipline with an integrated, evidence-based, and patient-centered approach. Therefore, this Research Topic contributes to building a more complex framework for modern clinical neuropsychology that better addresses the diverse and evolving needs of patients across the lifespan.

Given the specialized care required by these populations, standardized programs covering core competencies should be designed and implemented to create competent specialists in clinical neuropsychology, harmonizing curricular requirements across countries (6, 7). In this context, program modalities analogous to medical residency training may be highly suitable for promoting clinical neuropsychology as a recognized health specialty derived from clinical psychology.
